# Comparative and Evolutionary Analyses of *Meloidogyne* spp. Based on Mitochondrial Genome Sequences

**DOI:** 10.1371/journal.pone.0121142

**Published:** 2015-03-23

**Authors:** Laura Evangelina García, M. Virginia Sánchez-Puerta

**Affiliations:** 1 IBAM-CONICET and Facultad de Ciencias Agrarias, Universidad Nacional de Cuyo, Chacras de Coria, Mendoza, Argentina; 2 Facultad de Ciencias Exactas y Naturales, Universidad Nacional de Cuyo, Mendoza, Mendoza, Argentina; James Hutton Institute, UNITED KINGDOM

## Abstract

Molecular taxonomy and evolution of nematodes have been recently the focus of several studies. Mitochondrial sequences were proposed as an alternative for precise identification of *Meloidogyne* species, to study intraspecific variability and to follow maternal lineages. We characterized the mitochondrial genomes (mtDNAs) of the root knot nematodes *M*. *floridensis*, *M*. *hapla* and *M*. *incognita*. These were AT rich (81–83%) and highly compact, encoding 12 proteins, 2 rRNAs, and 22 tRNAs. Comparisons with published mtDNAs of *M*. *chitwoodi*, *M*. *incognita* (another strain) and *M*. *graminicola* revealed that they share protein and rRNA gene order but differ in the order of tRNAs. The mtDNAs of *M*. *floridensis* and *M*. *incognita* were strikingly similar (97–100% identity for all coding regions). In contrast, *M*. *floridensis*, *M*. *chitwoodi*, *M*. *hapla* and *M*. *graminicola* showed 65–84% nucleotide identity for coding regions. Variable mitochondrial sequences are potentially useful for evolutionary and taxonomic studies. We developed a molecular taxonomic marker by sequencing a highly-variable ~2 kb mitochondrial region, *nad5-cox1*, from 36 populations of root-knot nematodes to elucidate relationships within the genus *Meloidogyne*. Isolates of five species formed monophyletic groups and showed little intraspecific variability. We also present a thorough analysis of the mitochondrial region *cox2-rrnS*. Phylogenies based on either mitochondrial region had good discrimination power but could not discriminate between *M*. *arenaria*, *M*. *incognita* and *M*. *floridensis*.

## Introduction

Mitochondria are subcellular organelles derived from an alpha-proteobacterial endosymbiont, wherein oxidative phosphorylation and other important biochemical functions take place [1]. Most of the genes of the original bacterial chromosome were lost or transferred to the nucleus, with a handful of genes remaining in animal mitochondrial genomes (mtDNAs) [2]. Animal mtDNAs share several features, including relatively constant gene content and order, maternal inheritance, reduced recombination rate, and high evolutionary rate. This molecule is central in disease, apoptosis and ageing studies, in genetic and genomic analyses, and in developing genetic markers for molecular systematics [3]. Animal mtDNAs are generally small and circular molecules containing 12–13 protein-coding genes for enzymes of the oxidative phosphorylation pathway, 22 transfer RNA-coding genes (tRNAs), and 2 ribosomal RNA-coding genes [2]. This gene content is variable only in a few groups of metazoans, such as some nematodes that lack the gene *atp8* [3], a bivalve that also lacks *atp8* and has an extra tRNA gene [4], and cnidarians that have lost nearly all tRNAs genes [5]. Usually, no introns are present in animal mtDNAs and intergenic sequences are small, although one large non-coding region with control elements is found in some metazoan species [6]. To date, more than 100 complete mtDNA sequences of the phylum Nematoda have been published. A phylum-wide comparison revealed that nematode mtDNAs are characterized by a very rapid rate of mitochondrial gene rearrangement and are smaller in size than those of other metazoan groups, varying in size from 13 kb to 15 kb with some exceptions as *Romanomermis culicivorax* (26 kb), *Pratylenchus vulnus* (21 kb) *Hexamermis agrotis* (24 kb) [2,3,7,8,9].

Root-knot nematodes (genus *Meloidogyne*, infraorder Tylenchomorpha, suborder Tylenchina, order Rhabditida, class Chromadorea) [10] are plant parasites, comprising more than 100 species, in addition to several described races [11] and rank first based on a list of scientifically and economically important plant parasitic nematodes [12]. Their common name refers to typical galls on the roots of host plants that reduce the uptake of water and nutrients resulting in lower crop productivity [13]. The genus *Meloidogyne* includes the most widespread and economically damaging nematodes worldwide: *M*. *incognita*, *M*. *javanica*, *M*. *arenaria*, *M*. *hapla*, *M*. *chitwoodi* and *M*. *enterolobii* [14]. Accurate identification of *Meloidogyne* species is critical for effective crop management. Traditionally, species diagnosis was done based on morphological characters, host range and esterase isozyme electrophoresis and were later combined with DNA-based methods [15]. These molecular techniques were initially based on analyses of DNA fragments and sequencing of the nuclear rDNA operon and became obsolete or less effective with the increasing description of new species [16]. Mitochondrial sequences were proposed as an alternative for precise identification of *Meloidogyne* species, to study intraspecific variability and to follow maternal lineages [17]. Mitochondrial markers have been used in the past, but with a different fragment than in this current study [17,18,19,20].

To gain insight into the evolutionary patterns, sequence diversity and potential taxonomic use of the mitochondrial genome of the genus *Meloidogyne*, we characterized the mtDNAs of three root-knot nematodes: a facultative meiotic parthenogenetic strain of *M*. *hapla* and *M*. *floridensis* and a strain of the obligate mitotic parthenogenetic *M*. *incognita*, taking advantage of genomic projects that focused on these species. We undertook detailed genomic comparisons among them and with mtDNAs of *Meloidogyne* spp. recently published [21,22,23]. Based on genomic alignments of *Meloidogyne*, we identified a 2 kb variable mitochondrial marker and performed phylogenetic analyses to aid in *Meloidogyne* species identification and evolution and to elucidate relationships within the genus.

## Materials and Methods

### Mitochondrial genome annotation

The nuclear genomes of three species of root-knot nematodes, *M*. *floridensis* [24], *M*. *hapla* [25], and *M*. *incognita* [26] have been reported and mitochondrial sequences were made available in public sequence databases as part of these genome sequencing projects but have not been yet assembled or analyzed. Sequence similarity searches were performed with BLASTn [27] against the GenBank databases nr, WGS (whole-genome shotgun), and EST (expressed sequence tag), restricted to the genus *Meloidogyne* without filtering low complexity regions and using the mtDNA of several Nematoda as the query sequence. A total of 17 contigs of the root-knot nematodes *M*. *floridensis*, *M*. *hapla* and *M*. *incognita* had similarity to mitochondrial genes (S1 Table). Mitochondrial fragments of each species were assembled based on overlapping sequences and by amplification and sequencing of one joining fragment. We designed primers to link two mitochondrial contigs of *M*. *hapla* ABLG01002800 and ABLG01002664 (S1 Table): Mh32800R (5`-AGAGTGAATTGGTAAGAGG-3`) and Mh12664F (5`-CGGTAACCAAAAACCTCCAAGC-3`). We amplified and sequenced a product of 1500 bp using DNA of *M*. *hapla* 7J2 (S2 Table). It is not possible to rule out the possibility that some of these mitochondrial sequences belong to NUMTs in the nuclear genome. However, the lack of nuclear sequences in the fragments containing mitochondrial genes suggests that those DNA fragments reside in the mitochondria.

Open reading frames of the mitochondrial sequences of *M*. *floridensis*, *M*. *hapla* and *M*. *incognita* were recognized with Sequencher 5.2.2 (Genes Codes Corporation) using the invertebrate mitochondrial genetic code. Each putative protein-coding gene was used as query for BLASTx searches against GenBank protein databases to identify them. Ribosomal RNA genes (rRNAs) were recognized by comparison to other rRNAs of nematodes using BLASTn [27]. The tRNA genes were identified using tRNAscan-SE search server [28] or by similarity to those reported in other *Meloidogyne* species. Secondary structures of tRNAs were predicted with tRNAscan-SE.

Gene boundaries, i.e. start and stop codons, are generally predicted based on the presence of a canonical start codon followed by a standard stop codon. When non-universal genetic codes or non-canonical stop codons are involved, as reported for animal mtDNAs [3], gene boundaries are less obvious and several start or stop codons may be available. A disparity in the criterion for establishing gene boundaries was observed for several genes in *Meloidogyne* mtDNA, even between two strains of the same species [21,23]. In this study, start codons were set at the first start codon available (including non-universal ones) that had the minimum overlap with an upstream gene. Complete universal stop codons (TAA or TAG) were selected even when they overlapped with a downstream gene.

### Analyses of mitochondrial sequences

Pairwise comparisons of protein, rRNA, and tRNA genes of *M*. *chitwoodi*, *M*. *floridensis*, *M*. *graminicola*, *M*. *hapla* and *M*. *incognita* were done in MEGA6 [29]. Codon usage of protein-coding genes and nucleotide composition were calculated with the software SMSv2 (Sequence Manipulation Suite) [30]. Whole-genome alignments of *Meloidogyne* spp. mitochondrial genomes were performed with the VISTA pipeline infrastructure [31]. Tandem repetitive elements were identified using Tandem Repeat Finder software [32].

### PCR amplification and sequencing of a mitochondrial region

DNA samples of 28 populations of 5 *Meloidogyne* species were kindly provided by Andrea Skantar (USDA) (S2 Table). DNA of a *M*. *arenaria* isolate from Argentina was extracted as reported previously [33]. We amplified and sequenced the mitochondrial region between the genes *nad5* and *cox1* from different *Meloidogyne* species and isolates using the following primers: *Mmt5* (5`-GGTTTAATTGGTGGTTTTGG-3`), *Mmt2rc* (5`-TGTCCTCAAACTAAACAACC-3`), *Mmt9* (5`-TTGGTTGATTGGTGAAAGC-3`), and *Mmt4rc* (5`-AAACCACCAATTAAACCAGG-3`). Primers were designed with the software Primaclade [34] based on a sequence alignment of available *Meloidogyne* species. PCR conditions included an initial denaturing step at 94°C for 2 min, followed by 35 cycles of 94°C for 30 seconds, 45°C for 30 seconds and 72°C for 1 min; followed by a final extension step at 72°C for 8 min. Amplified fragments were sequenced by Sanger sequencing with Applied Biosystems 3730XL. Sequences were deposited in GenBank (accession numbers KM491188-KM491216).

### Phylogenetic analyses

Three mitochondrial nucleotide data sets were constructed and analyzed. One consisted of 8 protein-coding genes (*cox1*, *cox2*, *cox3*, *cob*, *nad2*, *nad3*, *nad4* and *nad5*) from 43 diverse nematode species that were retrieved from GenBank. The 8 protein-coding genes were concatenated in a single nucleotide data set of 4,920 bp. The second data set included sequences of the *nad5-cox1* region from 36 populations of 8 species of *Meloidogyne* (S2 Table). The third data set consisted of mitochondrial sequences of the *cox2-rrnS* region from 151 isolates of 20 *Meloidogyne* species that were retrieved from GenBank. Nucleotide sequences were manually aligned with MacClade 4.07 [35]. Alignment regions for which positional homology could not be determined with confidence by visual inspection were excluded. Alignments and trees have been deposited in Treebase (study S17102).

Maximum Likelihood (ML) phylogenetic analyses were performed with Garli 0.951 [36] under the General Time Reversible model with parameters for invariable sites and gamma-distributed rate heterogeneity. The GTR substitution model was chosen by using Modeltest for each data set [37]. Ten independent runs were conducted using either the automated stopping criterion or for up to 5,000,000 generations to ensure convergence to a similar topology and likelihood score. A hundred ML bootstrap replicates were performed. Maximum Parsimony (MP) analyses were conducted using PAUP* 4.0 [38]. The search strategy involved tree-bisection reconnection branch swapping, MulTrees option in effect. To assess node support, MP bootstrap analyses were performed using 1,000-replicate heuristic search.

## Results and Discussion

### Characterization of the mitochondrial genomes of *M*. *incognita*, *M*. *floridensis*, and *M*. *hapla*

**Fig 1:**
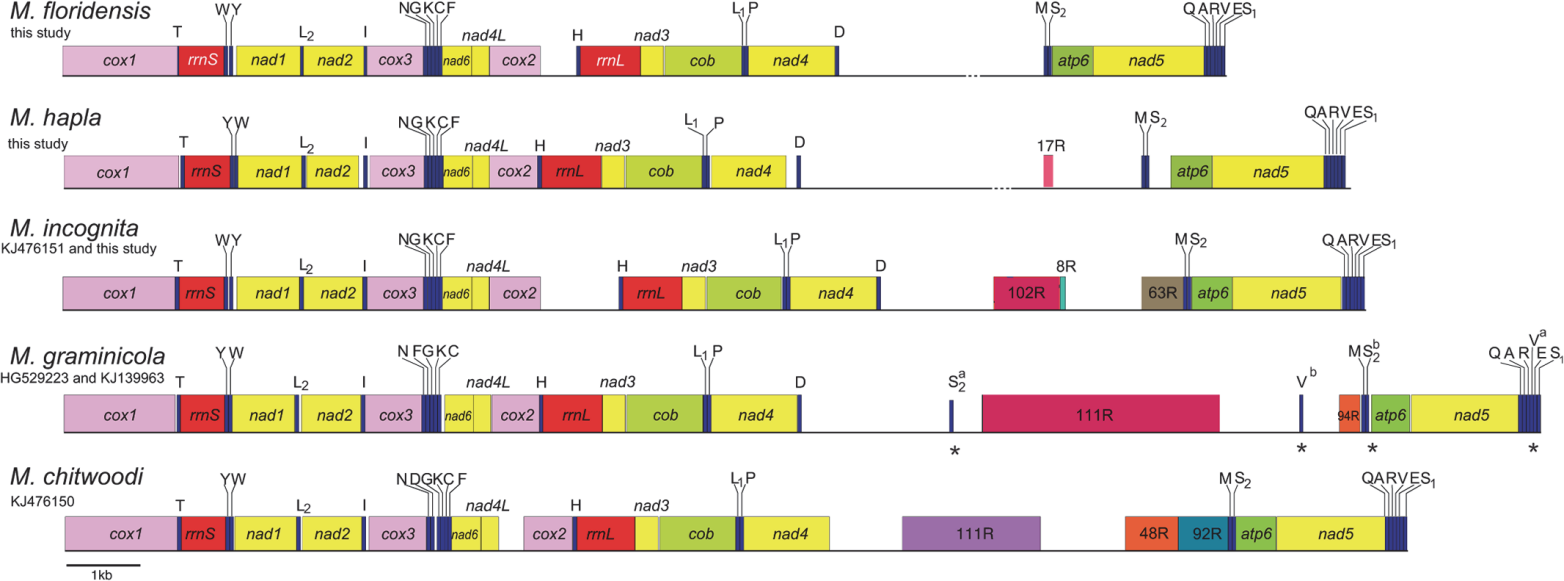
Linear maps of the mitochondrial genomes of five *Meloidogyne* species. The maps were drawn to scale. Dots interrupting the main genomic line indicate unknown sequences. All genes are encoded on the same strand of the mtDNA. Protein and rRNA genes have standard nomenclature. tRNA genes are designated by single-letter abbreviation. Two tRNAs for Leucine (L) and Serine (S) are present: L_1_ (anticodon UAG), L_2_ (UAA), S_1_ (UCN) and S_2_ (AGN). S^a^, V^a^, S^b^, and V^b^ correspond to tRNA genes annotated differently in two previous studies (S^a^ and V^a^ in GenBank KJ139963 [23]; S^b^ and V^b^ in GenBank HG529223 [21]). Tandem repeats are represented by boxes labeled by the repeat length in kb followed by “R”.

The mtDNA of *M*. *incognita* assembled as a circular molecule of 17,985–18,332 bp, depending on the number of 63-bp repeats (shown linearized in Fig. 1). The incomplete mitochondrial genomes of *Meloidogyne hapla* and *M*. *floridensis* assembled as single molecules of 17,355 bp and 15,811 bp respectively (Fig. 1, Table 1). The total length of the latter genomes is unknown due to missing data in the long intergenic region between the genes *nad4* and *atp6* (Fig. 1). Complete mitochondrial genomes of three *Meloidogyne* spp. have been described with similar genome length (Table 1, Fig. 1). Also, the size of the mtDNA of *M*. *javanica* (20.5 kb) has been reported [39]. A comparison to other animal mtDNAs revealed that *Meloidogyne* mtDNAs fall within the size range of Metazoa (8 kb to 48 kb)[6,40].

**Table 1:** Comparisons between sequenced mitochondrial genomes of the genus *Meloidogyne*.

	*M*. *floridensis* (isolate 5)	*M*. *hapla* (isolate VW9)	*M*. *incognita* (isolate Morelos)	*M*. *incognita* (isolate NCMI4)	*M*. *chitwoodi* (isolate CAMC2)	*M*. *graminicola* (isolate from Batangas)	*M*. *graminicola* (isolate from Hainan)
Genome size	>15,811 bp	>17,355 bp	17,985–18,332 bp^b^	17,662–19,100 bp	18,201 bp	20,030 bp	19,589 bp
AT content (%)	83.5%	81.4%	83.3%	83.0%	85.0%	84.3%	83.5%
Protein-coding sequence length (%)	nd^c^	nd^c^	53–54%	51–54%	49.4%	49.0%	50.6%
Repeats^a^	no repeats found	17 bp (7.6)	102 bp (12.8)	102 bp (19–24)	111 bp (16.9)	111 bp (29)	111 bp (25)
			8 bp (9.4)	8 bp (10.4)	48 bp (15.6)	94 bp (3)	94 bp (3)
			63 bp (7.9–13.4)	63 bp (9)	92 bp (8.1)		
References	this study			[15]		[14]	[16]

^a^ Numbers in parentheses indicate the copy-number of each repeat.

^b^ The genome size depends on the number of copies of the R63 repeat, which varies from 7 to 13 in the genomic assembly.

^c^ Not determined because the total genome length is unknown.

The mitochondrial genomes of the five *Meloidogyne* spp. available, *M*. *hapla*, *M*. *incognita*, *M*. *graminicola*, *M*. *chitwoodi* and *M*. *floridensis*, shared several features (Fig. 1). They contained 12 protein-coding genes (*atp8* was missing), 2 rRNA-coding genes (*rrnS* and *rrnL*), and 22 tRNA-coding genes (Fig. 1). Loss of the gene *atp8* has been related to the parasitic lifestyle in other organisms [41]; however, both free-living and parasitic chromadorean nematodes lack this gene [3].

We identified 22 discrete sequences (ranging from 49 to 59 bp) predicted to fold into secondary structures of tRNAs in the mtDNAs of *M*. *floridensis*, *M*. *hapla*, and *M*. *incognita*, respectively. In each of the species, 20 tRNAs had the predicted T-arm-lacking structure [5] and two putative tRNA genes folded into different secondary structures, such as the genes tRNA-S_1_(UCN) and tRNA-S_2_(AGN) that had a TyC arm and loop but not the DHU arm.

All genes in *Meloidogyne* mtDNAs were encoded on the same strand, with very few and often short intergenic sequences (Fig. 1; S3 Table). This asymmetry in transcriptional architecture is typical of chromadoreans, while enopleans and most animal species have circular mitochondrial genomes with coding genes on both strands [42,43]. The nucleotide composition of *Meloidogyne* spp. mtDNAs showed a large bias towards AT (Table 1), similar to that of their nuclear genomes [24]. In general, mitochondrial genomes of the phylum Nematoda showed a trend for AT richness, ranging from 66% to 85%. In addition, a bias towards T and G content in the coding strand was observed in the mtDNAs of *M*. *floridensis*, *M*. *hapla* and *M*. *incognita*, as reported for other nematodes [23,44,45,46].

Start and stop codons of protein-coding genes in *Meloidogyne* mtDNAs were often non-universal. Putative start codons for the 12 mitochondrial protein-coding genes in *M*. *hapla*, *M*. *incognita* and *M*. *floridensis* were ATA, ATG, ATT, TTA, TTG; stop codons were TAA and TAG (S3 Table). Some of these start codons are non-universal and have been previously reported for nematode mitochondrial genomes [21,22,23,47,48,49]. In addition, the termination codon TGA encoded tryptophan, among other deviations from the universal genetic code in nematode mitochondria [50].

### Phylogeny of Nematoda based on mitochondrial sequences

We performed a Maximum Likelihood phylogenetic analysis based on nucleotide sequences of eight mitochondrial protein-coding genes (4,920 bp) from 43 species of the phylum Nematoda, including 5 *Meloidogyne* spp. and 8 taxa of the class Enoplea as outgroups (S1 Fig.). Mitochondrial genes could resolve most phylogenetic relationships within the phylum Nematoda and these were consistent with previous studies based on mitochondrial [7,8,22] and nuclear genes [51,52]. *Meloidogyne* spp. grouped together as sister to *Pratylenchus vulnus* within the infraorder Tylenchomorpha with high bootstrap support (S1 Fig.). Within the clade of *Meloidogyne*, *M*. *incognita* + *M*. *floridensis* were sister to *M*. *hapla* with great bootstrap support (BS = 100%), and these were sister to *M*. *chitwoodi* + *M*. *graminicola*. These findings agree with previous studies based on the nuclear gene 18S rRNA [53].

### Gene order in nematode mitochondrial genomes

Protein and rRNA gene order was identical within the genus *Meloidogyne* (Fig. 1), but different to other members of the infraorder Tylenchomorpha and any other nematode (S1 Fig.). Furthermore, the location of the tRNA genes was unique for four species of *Meloidogyne*, except for *M*. *incognita* and *M*. *floridensis* that were identical in gene order (Fig. 1). Differences occurred mainly in two blocks of tRNAs; those located between *rrnS* and *nad1* and the group found between the genes *cox3* and *nad6*.

Identification of tRNA genes is controversial in nematode mitochondrial genomes given that tRNAs are short, divergent and have atypical structures [54]. As a result, we noticed discrepancies in the prediction of tRNA genes in published *Meloidogyne* mtDNAs. For example, there was no consensus between the tRNA annotations of the highly similar (see below) mitochondrial genomes of two strains of *M*. *graminicola* (asterisks in Fig. 1) using two different tRNAs prediction programs [21,23].

Nematode mtDNAs, including those of root-knot nematodes, showed mitochondrial evolutionary dynamics that differ from most metazoan [55]. For example, mitochondrial genomic rearrangements within the phylum Nematoda were significantly higher than those of other animal groups [3]. A previous study found 25 different gene rearrangements (GA) in 62 mitochondrial genomes of nematodes, considering the position of tRNAs; of them, the most common was GA3, which was shared by 32 nematode species [8]. Within the infraorder Tylenchomorpha, several mtDNAs have recently become available. *Heterodera glycines* and *Pratylenchus vulnus* [7,46] showed two different gene arrangements to those already described (indicated as GA26 and GA27 in S1 Fig.). The mtDNAs of *Meloidogyne* spp. added four novel gene rearrangements to the list of described GA in Nematoda (GA28-GA31; S1 Fig.). The genus *Meloidogyne* (and the infraorder Tylenchomorpha as a whole) are particularly variable in gene order in comparison to other lineages of Nematoda, along with the highly re-arranged enopleans [43,56], and the genus *Onchocerca* [49,57].

### Non-coding regions in *Meloidogyne* mtDNAs

The mtDNA of *M*. *hapla* was highly compact and presented 3 intergenic regions larger than 100 bp (Fig. 1): *nad4*-*trnD* (144 bp), *trnS*-*atp6* (290 bp), and *trnD-trnM* (>4,596 bp, the major non-coding region). In the latter, a tandem repeat of 17 bp (17R) was found (Table 1). Previously, three tandem repeats of 102, 8 and 63 bp were reported for *M*. *hapla* [39], but subsequent reports [19] and this study did not find such sequences in *M*. *hapla*.

The mtDNA of *M*. *floridensis* had only 2 intergenic regions >100 bp: *cox2*-*trnH* (535 bp) and *trnD-trnM* (>2,774 bp) and no tandem repeats were identified (Fig. 1). *M*. *incognita* mtDNA also had 2 intergenic regions in the same locations as in *M*. *floridensis*: *cox2*-*trnH* (1,063 bp) and *trnD-trnM* (4,420–5,910 bp, depending on the number of repeats). Within the latter, 102-bp (102R) and 63-bp (63R) tandem repeats were found in *M*. *incognita* (Fig. 1). The 63R had also been reported for *M*. *arenaria*, *M*. *javanica* [39], and *M*. *enterolobii* [19]. Different copy-numbers of the 63R were described for a single individual (heteroplasmy) or among populations of the same species for *M*. *javanica*, *M*. *arenaria* and *M*. *incognita* [19,39]. The 63R region has been explored as a molecular marker but the results were difficult to interpret and its use was questionable [19,20].

The *cox2*-*rrnS* region has been largely studied and used in molecular systematics [17,18,19,58,59] because it has indels and polymorphisms useful to differentiate species of the genus *Meloidogyne*. Until recently, it was possible to identify the major species of *Meloidogyne* by PCR amplification and digestion of this fragment, or by DNA sequencing of this region. Lately, the analysis of the *cox2-rrnS* in additional *Meloidogyne* species uncovered limitations in the taxonomic use of this marker (see below).

The largest intergenic region in all *Meloidogyne* spp. mtDNAs was found between the protein-coding genes *nad4* and *atp6* and included 2–5 tRNA-coding genes (Fig. 1). This region also contained tandem repeats in most species, but the repeats were not homologous with a few exceptions (Fig. 1). *M*. *graminicola* had a 111-bp (111R) tandem repeat that was similar to the 102-bp (102R) tandem repeat of *M*. *incognita*, but different to the 111-bp (111R) tandem repeat of *M*. *chitwoodi*. Also, *M*. *chitwoodi* had a 48-bp (48R) tandem repeat that aligned with the 94-bp (94R) repeat of *M*. *graminicola* (Fig. 1).

### Variability of mitochondrial genomes within a single *Meloidogyne* species

The mtDNAs of 2 strains of *M*. *incognita* (this study and [15]) and 2 strains of *M*. *graminicola* [21,23] are now available for intraspecific comparisons. Variation in the copy-number of tandem repeats and the presence of indels in intergenic regions are responsible for the differences in genome length within each species (Table 1). A pairwise comparison between the mtDNA of the 2 strains of *M*. *incognita* and *M*. *graminicola*, respectively, indicated that they shared the same gene content and order (not considering the discrepancy in tRNA annotation of the *M*. *graminicola* mtDNAs shown in Fig. 1 given that the putative tRNAs were present in both strains).

Mitochondrial sequences between the strains of *M*. *incognita* were extremely similar showing an identity of 99.2% (excluding gaps) with only a few substitutions and indels across coding and non-coding regions (S2-A Fig.). A pairwise comparison between the mtDNAs of both strains of *M*. *graminicola* showed that they were almost identical (99.9% identity, excluding gaps) with very few polymorphisms in the coding regions and most substitutions clustered within the *trnD-trnV* non-coding region (S2-B Fig.). The closer geographic distance between the *M*. *graminicola* strains (China and Philippines) in comparison to the two isolates of *M*. *incognita* (Mexico and France) could relate to the higher mtDNA similarity observed within *M*. *graminicola*. It is also possible that the mitochondrial sequence of *M*. *incognita* strain Morelos were contaminated with NUMTs, showing more substitutions than the actual mtDNA residing in the mitochondria [60,61].

### Variability of mitochondrial genomes among *Meloidogyne* spp.

**Fig 2:**
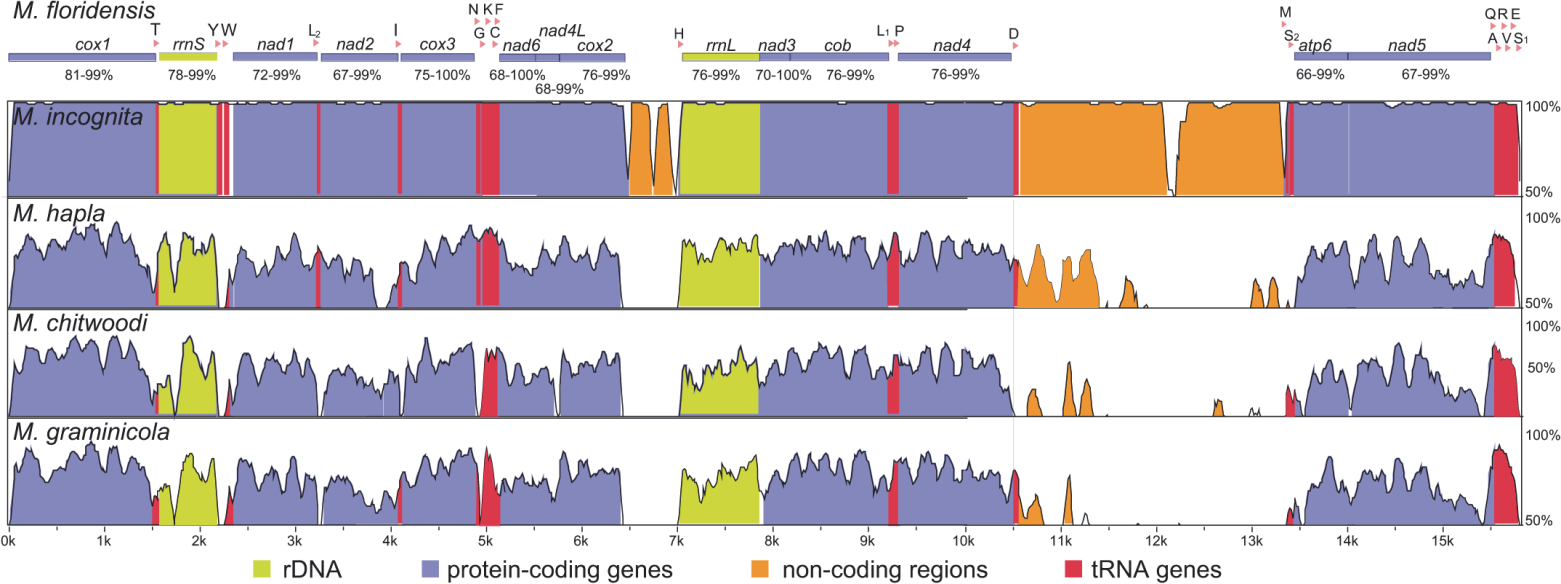
Pairwise comparisons between the mitochondrial genome of *M*. *floridensis* (this study) and that of *M*. *incognita* (KJ47615), *M*. *hapla* (this study), *M*. *chitwoodi* (KJ476150) and *M*. *graminicola* (KJ139963) using the program VISTA. The Y-axis represents the sequence identity (50–100%). Protein (in violet) and rRNA (in yellow) genes are indicated in the standard nomenclature. tRNA genes (in red) are designated by a single-letter abbreviation. The range of identity (%) of protein-coding genes among *Meloidogyne* spp. is shown at the top.

The mtDNA of *M*. *floridensis* was compared to that of other *Meloidogyne* spp. with the program VISTA (Fig. 2). *M*. *floridensis* and *M*. *incognita* mtDNAs were strikingly similar across the genome with an identity of 97–100% for all coding regions. The genes *cox3*, *nad6* and *nad3*, along with 19 tRNAs, were identical in both species while the other genes showed 1–7 substitutions/gene. This result is in agreement with recent evidence showing that *M*. *incognita* is a double hybrid of *M*. *floridensis* and another species [24]. Comparisons to *M*. *hapla*, *M*. *graminicola* and *M*. *chitwoodi* revealed the presence of conserved genic regions and highly divergent (including some non-homologous) intergenic sequences (Fig. 2). The same is true for pairwise comparisons between the other *Meloidogyne* species.

The alignments of each mitochondrial gene from *M*. *floridensis*, *M*. *incognita*, *M*. *hapla*, *M*. *graminicola* and *M*. *chitwoodi* showed >65% nucleotide identity for most coding regions (Fig. 2; S3 Table). Overall comparisons within the genus *Meloidogyne* indicated that the most conserved protein-coding gene was *cox1* (81–99% identity; Fig. 2). Genes encoding rRNAs had an identity of 78–99% (*rrnS*) and 76–99% (*rrnL*). In contrast, tRNA genes had a wider range of identity (47–100%) within the genus, being *trnS* and *trnV* the most divergent tRNAs (S3 Table).

### Analysis of mitochondrial regions for taxonomic use

**Fig 3:**
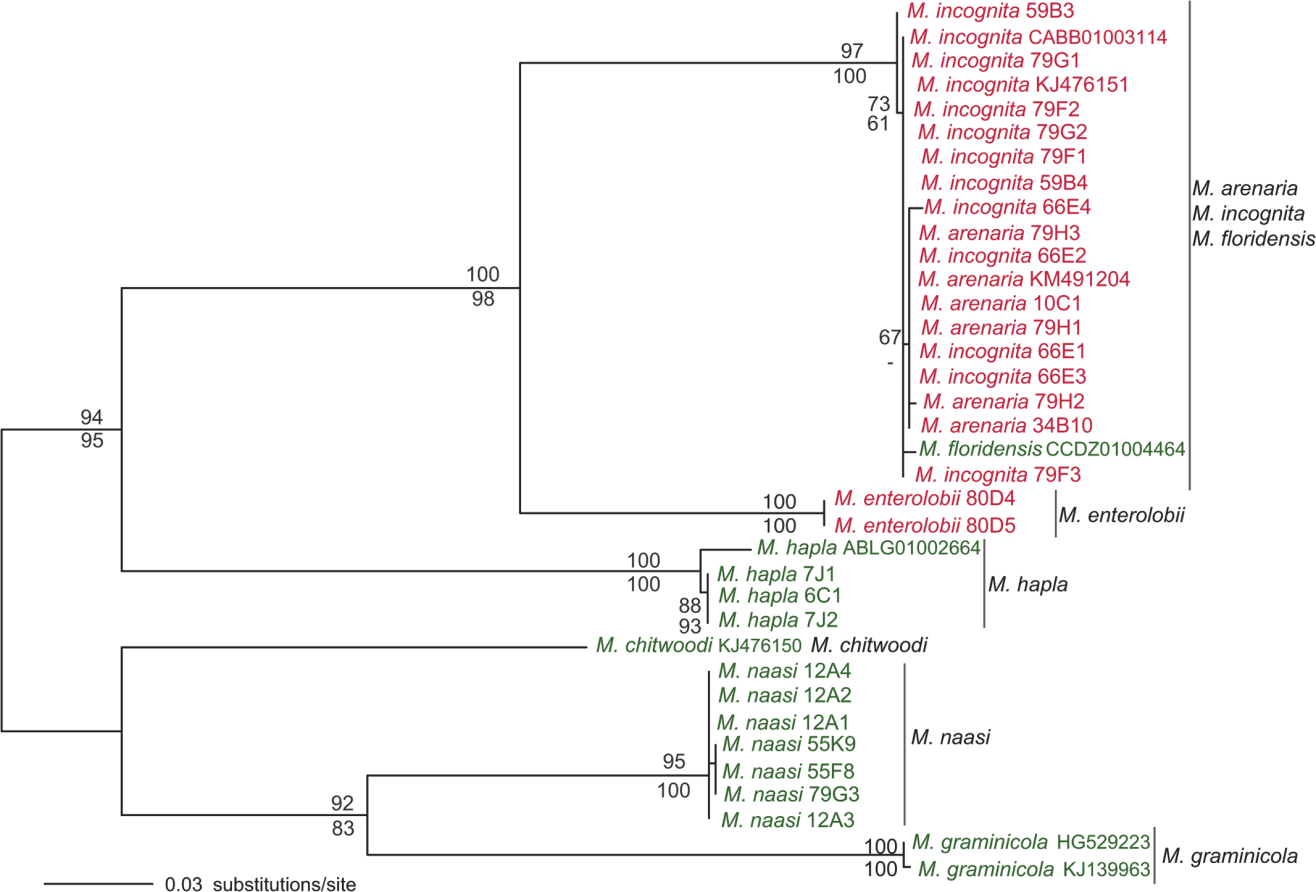
Maximum Likelihood phylogenetic tree of *Meloidogyne* spp. based on the *nad5*-*cox1* mitochondrial region. The tree was rooted according to Holterman *et al*. [53]. Numbers above and below branches represent ML and MP bootstrap support values >50%, respectively. Species names in red and green indicate mitotic and meiotic parthenogenetic species, respectively.

Variable intergenic mitochondrial sequences are potentially useful for phylogenetic studies and species identification. Based on mitochondrial genomic comparisons (i.e. Fig. 2), we identified a highly variable ~2 kb mitochondrial segment starting on the gene *nad5* through the middle of the gene *cox1*, including 6 tRNAs and intergenic regions. We selected this region because it showed interspecific variability and it was easy to amplify by PCR from a wide range of species given the presence of highly conserved flanking sequences. The *nad5-cox1* region was sequenced and analyzed in 36 populations of 8 *Meloidogyne* spp. (S2 Table). The overall topologies of the ML and MP phylogenetic trees were almost identical (Fig. 3).

Isolates of each species formed monophyletic groups with high bootstrap support (95–100%), except for populations of *M*. *arenaria*, *M*. *incognita* and *M*. *floridensis* that grouped in a single clade but were not separated by species (Fig. 3). The few polymorphisms observed in this heterogeneous group fell within the range of intraspecific variability. Within each of the monophyletic species in the tree, sequences were highly conserved with very few or no polymorphisms. This limited intraspecific variability (Fig. 3) has been reported for parthenogenetic nematodes [17,62] and it is now extended to sexual species (Fig. 3). The mitochondrial genomic comparisons between the 2 strains of *M*. *incognita* and *M*. *graminicola* (S2 Fig.) also agreed with this statement. Phylogenetic relationships observed among the sampled species (Fig. 3) were congruent with previous phylogenies based on the nuclear 18S rRNA [63] or the mitochondrial region *cox2-rrnS*.

Besides the mitochondrial marker *nad5-cox1*, a single mitochondrial region, *cox2-rrnS*, has been extensively analyzed within the genus *Meloidogyne* [17,18,19,20]. Phylogenetic trees based on this marker had good discriminating power but relationships among several species of root-knot nematodes were unresolved and several isolates could not be identified at the species level [58,64,65,66]. A phylogeny including all *cox2-rrnS* sequences available in public databases for *Meloidogyne* species (S3 Fig.) showed that *M*. *graminis*, *M*. *marylandi*, *M*. *hapla*, *M*. *partityla*, *M*. *haplanaria*, *M*. *enterolobii*, *M*. *naasi* and *M*. *graminicola* could be separated based on a number of shared substitutions that took place since each species diverged. In contrast, some species had highly similar *cox2-rrnS* sequences and could not be differentiated. For example, *M*. *fallax* was paraphyletic with respect to *M*. *chitwoodi* with almost identical sequences (S3 Fig.). The greatest unresolved clade included the major and most economically damaging *Meloidogyne* spp, along with other minor root-knot nematodes (S3 Fig.). These 10 species formed a monophyletic clade with highly similar *cox2-rrnS* sequences and indels shared by different taxa, which prevented a clear distinction of species based on this marker.

These and previous studies based on nuclear or mitochondrial data [53,58,63,67] found that mitotic parthenogenetic *Meloidogyne* species (shown in red in Fig. 3 and S3 Fig.) were evolutionary distinct from the meiotic parthenogenetic ones (shown in green in Fig. 3 and S3 Fig.), with a few exceptions such as *M*. *floridensis* that is meiotic parthenogenetic and clusters within the first group (Fig. 3; S3 Fig.).

In addition to the results found with both mitochondrial markers, other studies also indicated that mitotic parthenogenetic species and *M*. *floridensis* are hard to distinguish using the nuclear regions ITS and 18S rDNA [33,53,65,68] and occasionally showed contradictory results between nuclear and mitochondrial phylogenies [17,58]. Species boundaries, especially for mitotic parthenogenetic nematodes, have been discussed [17] and interspecific hybridizations prior to the recent establishment of parthenogenesis were proposed [17,69]. A comparative analysis of gene families based on the complete nuclear genomes of *M*. *hapla* [25], *M*. *incognita* [26] and *M*. *floridensis* [24] suggested that *M*. *incognita* is a double-hybrid between *M*. *floridensis* and an unidentified species [24].

It remains challenging to distinguish most mitotic parthenogenetic *Meloidogyne* isolates and to identify the species, if they, indeed, belong to different phylogenetic species. Extensive studies that include a wide range of populations and species of *Meloidogyne* while comparing mitochondrial and nuclear sequences are needed to elucidate the complex evolution of this group. Single copy nuclear genes may aid in the identification of mitotic parthenogenetic species.

## Supporting Information

S1 Fig Maximum Likelihood phylogenetic tree based on nucleotide sequences of eight mitochondrial protein-coding genes (4,920 bp). Numbers above branches represent bootstrap support values >50%. The mtDNA gene arrangement (GA) according to Liu *et al*. [8] is shown next to each species name.
(PDF)

S2 Fig Pairwise comparisons between the mtDNA of two strains of *M*. *incognita* (A) and two strains of *M*. *graminicola* (B), respectively, using the program VISTA. The Y-axis represents the sequence identity (50–100%). Protein (in violet) and rRNA (in yellow) genes are indicated in the standard nomenclature. tRNA genes (in red) are designated by a single-letter abbreviation.
(PDF)

S3 Fig Maximum Likelihood (ML) phylogenetic tree of *Meloidogyne* spp. based on the *cox2*-*rrnS* mitochondrial region. Numbers above branches represent ML bootstrap values >50%. Species names in red, green or black indicate species with mitotic parthenogenesis, meiotic parthenogenesis or unknown reproductive strategy, respectively.
(PDF)

S1 Table GenBank accession numbers of *Meloidogyne* spp. with similarity to mitochondrial DNA. (PDF)

S2 Table Taxonomic information and source of the *Meloidogyne* isolates included in this study. (PDF)

S3 Table Mitochondrial genome organization of *M*. *hapla*, *M*. *floridensis* and *M*. *incognita*. (PDF)

## References

[1] Koonin EV. The origin and early evolution of eukaryotes in the light of phylogenomics. Genome Biol. 2010; 11: 1–12. 10.1186/gb-2010-11-5-209 PMC2898073 20441612

[2] Boore JL. Animal mitochondrial genomes. Nucleic Acids Res. 1999; 27: 1767–1780. 10101183 10.1093/nar/27.8.1767 PMC148383

[3] Hu M, Gasser RB. Mitochondrial genomes of parasitic nematodes-progress and perspectives. Trends parasitol. 2006; 22: 78–84. 16377245 10.1016/j.pt.2005.12.003

[4] Hoffmann RJ, Boore JL, Brown WM. A novel mitochondrial genome organization for the blue mussel, Mytilus edulis. Genetics. 1992; 131: 397–412. 1386586 10.1093/genetics/131.2.397 PMC1205014

[5] Wolstenholme DR. Animal mitochondrial DNA estructure and evolution. Int Rev Cytol. 1992; 141: 173–216. 1452431 10.1016/s0074-7696(08)62066-5

[6] Lavrov D. Mitochondrial Genomes in Invertebrate Animals In: Bell E, editor. Molecular Life Sciences: Springer New York; 2014 pp. 1–8.

[7] Sultana T, Kim J, Lee SH, Han H, Kim S, Min GS, et al Comparative analysis of complete mitochondrial genome sequences confirms independent origins of plant-parasitic nematodes. BMC Evol Biol. 2013; 13: 1471–2148. 10.1186/1471-2148-13-12 PMC3558337 23331769

[8] Liu HG, Shao R, Li JY, Zhou DH, Li H, Zhu XQ. The complete mitochondrial genomes of three parasitic nematodes of birds: a unique gene order and insights into nematode phylogeny. BMC Genomics. 2013; 14: 414 10.1186/1471-2164-14-414 23800363 PMC3693896

[9] Azevedo JL, Hyman BC. Molecular characterization of lengthy mitochondrial DNA duplications from the parasitic nematode Romanomermis culicivorax. Genetics. 1993; 133: 933–942. 8462851 10.1093/genetics/133.4.933 PMC1205410

[10] De Ley P, Blaxter ML. A new system for Nematoda: combining morphological characters with molecular trees, and translating clades into ranks and taxa. Nematol Monogr Persp. 2004; 2: 633–653.

[11] Karssen G. The plant parasitic nematode genus *Meloidogyne* Goldi, 1892 (Tylenchida) in Europe Leiden, Netherlands: Brill 2002.

[12] Jones JT, Haegeman A, Danchin EG, Gaur HS, Helder J, Jones MG, et al Top 10 plant-parasitic nematodes in molecular plant pathology. Mol Plant Pathol. 2013; 14: 946–961. 10.1111/mpp.12057 23809086 PMC6638764

[13] Abad P, Favery B, Rosso MN, Castagnone-Sereno P. Root-knot nematode parasitism and host response: molecular basis of a sophisticated interaction. Mol Plant Pathol. 2003; 4: 217–224. 10.1046/j.1364-3703.2003.00170.x 20569382

[14] Sasser JN, Eisenback JD, Carter CC, Triantaphyllou AC. The international *Meloidogyne* project—its goals and accomplishments. Ann Rev Phytopathol. 1983; 21: 271–288.

[15] Hunt D, Handoo Z. Taxonomy, identification and principal species In: Perry RN, Moens M, Starr JL, editors. Root- knot nematodes. CABI, Wallingford; 2009 pp. 55–97.

[16] Carneiro R, Correa V, Almeida MR, Gomes AC, Mohammad Deimi A, Castagnone-Sereno P, et al *Meloidogyne luci* n. sp. (Nematoda: Meloidogynidae), a root-knot nematode parasitising different crops in Brazil, Chile and Iran. Nematology. 2014; 16: 289–301.

[17] Fargette M, Berthier K, Richaud M, Lollier V, Franck P, Hernandez A, et al Crosses prior to parthenogenesis explain the current genetic diversity of tropical plant parasitic *Meloidogyne* species (Nematoda: Tylenchida). Infect Genet Evol. 2010; 10: 807–814. 10.1016/j.meegid.2009.04.013 19393769

[18] Powers TO, Harris TS. A polymerase chain reaction method for identification of five major *Meloidogyne* species. J Nematol. 1993; 25: 1–6. 19279734 PMC2619349

[19] Blok VC, Wishart J, Fargette M, Berthier K, Phillips M. Mitochondrial DNA differences distinguishing *Meloidogyne mayaguensis* from the major species of tropical root-knot nematodes. Nematology. 2002; 4: 773–781.

[20] Lunt DH, Whipple LE, Hyman BC. Mitochondrial DNA variable number tandem repeats (VNTR): utility and problems in molecular ecology. Mol Ecol. 1998; 7: 1441–1455. 9819900 10.1046/j.1365-294x.1998.00495.x

[21] Besnard G, Juhling F, Chapius E, Zedane L, Lhuillier E, Mateille T, et al Fast assembly of the mitochondrial genome of a plant parastic nematode (*Meloidogyne graminicola*) using next generation sequencing. C R Biol. 2014; 337: 295–301. 10.1016/j.crvi.2014.03.003 24841955

[22] Humphreys- Pereira DA, Elling A. Mitochondrial genomes of *Meloidogyne chitwoodi* and *Meloidogyne incognita* (Nematoda: Tylenchina): Comparative analysis, gene order and phylogenetics relationship with other nematodes. Mol Biochem Parasit. 2014; 194: 20–32. 10.1016/j.molbiopara.2014.04.003 24751670

[23] Sun L, Zhuo K, Lin B, Wang H, Liao J. The complete mitochondrial genome of *Meloidogyne graminicola* (Tylenchina): a unique gene arrangement and its phylogenetics implications. Plos one. 2014; 9: e98558 10.1371/journal.pone.0098558 24892428 PMC4043755

[24] Lunt DH, Kumar S, Koutsovoulos G, Blaxter ML. The complex hybrid origins of the root knot nematodes revealed through comparative genomics. PeerJ. 2014; 6. 10.7717/peerj.356 PMC4017819 24860695

[25] Opperman C, Birda D, Williamson V, Rokhsare D, Burkea M, Cohna J, et al Sequence and genetic map of *Meloidogyne hapla*: a compact nematode genome for plant parasitism. Proc Natl Acad Sci. 2008; 105: 14802–14807. 10.1073/pnas.0805946105 18809916 PMC2547418

[26] Abad P, Gouzy J, Aury JM, Castagnone-Sereno P, Danchin E, Deleury E, et al Genome sequence of the metazoan plant—parasitic nematode *Meloidogyne incognita* . Nat Biotechnol. 2008; 26: 909–915. 10.1038/nbt.1482 18660804

[27] Altschul SF, Gish W, Miller W, Myers EW, Lipman DJ. Basic local alignment search tool. J Mol Biol. 1990; 215: 403–410. 2231712 10.1016/S0022-2836(05)80360-2

[28] Schattner P, Brooks AN, Lowe TM. The tRNAscan-SE, snoscan and snoGPS web servers for the detection of tRNAs and snoRNAs. Nucleic Acids Research. 2005; 33: W686–W689. 15980563 10.1093/nar/gki366 PMC1160127

[29] Tamura K, Stecher G, Peterson D, Filipski A, Kumar S. MEGA6: Molecular Evolutionary Genetics Analysis version 6.0 Mol Biol Evol. 2013; 30 2725–2729. 10.1093/molbev/mst197 24132122 PMC3840312

[30] Stothard P. The sequence Manipulation Suite: JavaScript programs for analyzing and formating protein and DNA sequences. Biotechniques. 2000; 28: 1102–1104. 10868275 10.2144/00286ir01

[31] Frazer KA, Pachter L, Poliakov A, Rubin EM, Dubchak I. VISTA: Computational tool for comparative genomics. Nucleid Acid Res. 2004; 1: 273–279. 10.1093/nar/gkh458 PMC441596 15215394

[32] Benson G. Tandem repeat finder: a program to analyze DNA sequences. Nucleic Acid Res. 1999; 27: 573–580. 9862982 10.1093/nar/27.2.573 PMC148217

[33] García LE, Sanchez-Puerta MV. Characterization of a root-knot nematode population of *Meloidogyne arenaria* from Tupungato (Mendoza, Argentina). J Nematol. 2012; 44: 291–301. 23481918 PMC3547342

[34] Gadberry MD, Malcomber ST, Doust AN, Kellog EA. Primaclade-a flexible tool for find conserved PCR primers across multiple species. Bioinformatics. 2005; 21: 1263–1264. 15539448 10.1093/bioinformatics/bti134

[35] Madison DR, Madison WP. Macclade 4: Analysis of Phylogeny and Character solution. Sinauer Associates, Sunderland, M A 2000.

[36] Zwickl DJ. Genetic algorithm approaches for the phylogenetic analysis of large biological sequence dataset under the maximun likelihood criterion University of Texas, Austin 2006.

[37] Posada D, Crandall KA. ModelTest. Testing the modal of DNA substitution. Bioinformatics. 1998; 14: 817–818. 9918953 10.1093/bioinformatics/14.9.817

[38] Swofford DL (1998) PAUP*. Phylogenetic Analysis Using Parsimony (*and Other Methods). In: Associates S, editor. Suderland, Massachusetts.

[39] Okimoto R, Chamberline HM, Macfarlane JL, Wolstenholme DR. Repeat sequences sets in mitochondrial DNA molecules of root knot nematodes (*Meloidogyne)* nucleotide sequences, genome locations and potencial for host race identification Nucleic Acid Res. 1991; 19: 1619–1626. 2027769 10.1093/nar/19.7.1619 PMC333924

[40] Cameron SL, Yoshizawa K, Mizukoshi A, Whiting MF, Johnson KP. Mitochondrial genome deletions and minicircles are common in lice (Insecta: Phthiraptera). BMC Genomics. 2011; 12: 1471–2164. 10.1186/1471-2164-12-394 PMC3199782 21813020

[41] Hancock L, Goff L, Lane C. Red algae lose key mitochondrial genes in response to becoming parasitic. Genome Biol Evol. 2010; 2: 897–910. 10.1093/gbe/evq075 21081313 PMC3014286

[42] Bernt M, Bleidorn C, Braband A, Dambach J, Donath A, Fritzch G, et al A comprehensive analysis of bilaterian mitochondrial genomes and phylogeny. Mol Phylogenet Evol. 2013; 69: 352–354. 10.1016/j.ympev.2013.05.002 23684911

[43] Hyman BC, Lewis S, Tang S, Wu Z. Rampant gene rearrangement and haplotype hypervariation among nematode mitochondrial genomes. Genetica. 2011; 139: 611–615. 10.1007/s10709-010-9531-3 21136141 PMC3089818

[44] Jacob JE, Vanholme B, Van Leeuwen T, Gheysen G. A unique genetic code change in the mitochondrial genome of the parasitic nematode *Radopholus similis* BMC Res Notes. 2009. 10.1186/1756-0500-2-192 PMC2761399 19778425

[45] Hu M, Chilton NB, Gasser RB. The mitochondrial genome of *Strongyloides stercoralis* (Nematoda)—idiosyncratic gene order and evolutionary implications. Int J Parasitol. 2003; 33: 1393–1408. 14527522 10.1016/s0020-7519(03)00130-9

[46] Gibson T, Farrugia D, Barret J, Chitwood DJ, Rowe J, Subottin S, et al The mitochondrial genome of the soybean cyst nematode, *Heterodera glycines* . Genome Biol. 2010; 54: 565–564. 10.1139/g11-024 21745140

[47] Li MW, Lin RQ, Song H, Q., Wu XY, Zhu Q, X. The complete mitochondrial genomes of three *Toxocara* species of human and animal health significance. BMC Genomics. 2008: e224. 10.1186/1471-2164-9-224 PMC2396643 18482460

[48] Kim K-H, Eom KS, Park J-K. The complete mitochondrial genome of *Anisakis simplex* (Ascaridida: Nematoda) and phylogenetic implications. Int J Parasitol. 2006; 36: 319–328. 16442542 10.1016/j.ijpara.2005.10.004

[49] McNulty SN, Mullin AS, Vaughan JA, Tkach VV, Weil GJ, Fischer PU. Comparing the mitochondrial genomes of *Wolbachia*-dependent and independent filarial nematode species. BMC Genomics. 2012; 13: 1471–2164. 10.1186/1471-2164-13-145 PMC3409033 22530989

[50] Watanabe K. Unique features of animal mitochondrial translation system. Prc Jpn Acad Ser B Phys Biol Sci. 2010; 86: 11–39. 20075606 10.2183/pjab.86.11 PMC3417567

[51] Blaxter M, De Ley P, Garey JR, Liu LX, Scheldeman P, Vierstraete A, et al A molecular evolutionary framework for the Phylum Nematoda. Nature. 1998; 392: 71–75. 9510248 10.1038/32160

[52] Holterman M, van der Wurff A, van den Elsen S, van Megen H, Bongers T, Holovachov O, et al Phylum-wide analysis of ssu rDNA reveals deep phylogenetic relationships among nematodes and accelerated evolution toward crown clades. Mol Biol Evol. 2006; 23: 1792–1800. 16790472 10.1093/molbev/msl044

[53] Holterman M, Karssen G, van den Elsen S, van Megen H, Bakker J, Helder J. Small subunit rDNA- based phylogeny of the Tylenchida sheds light on relationship among some high-impact plant parasitic nematodes and the evolution of plant feeding. Phytopathology. 2009; 99: 227–235. 10.1094/PHYTO-99-3-0227 19203274

[54] Wende S, Platzer EG, Juhling F, Putz J, Florentz C, Stadler PF, et al Biological evidence for the world's smallest tRNAs. Biochimie. 2014; 100: 151–158. 10.1016/j.biochi.2013.07.034 23958440

[55] Gissi C, Iannelli F, Pesole G. Evolution of the mitochondrial genome of Metazoa as exemplified by comparison of congeneric species. Heredity. 2008; 101: 301–320. 10.1038/hdy.2008.62 18612321

[56] Powers TO, Harris TS, Hyman BC. Mitochondrial DNA Sequence Divergence among *Meloidogyne incognita*, *Romanomermis culicivorax*, *Ascaris suum*, and *Caenorhabditis elegans* . J Nematol. 1993; 25: 564–572. 19279810 PMC2619415

[57] Keddie EM, Higazi T, Unnasch TR. The mitochondrial genome of *Onchocerca volvulus*: sequence, structure and phylogenetic analysis. Mol Biochem Parasitol. 1998; 95: 111–127. 9763293 10.1016/s0166-6851(98)00102-9

[58] Tigano MS, Carneiro R, Jeyaprakash A, Dickson DW, Adams BJ. Phylogeny of *Meloidogyne* spp. based on 18s rDNA and the intergenic region of mitochondrial DNA sequences. Nematology. 2005; 7: 851–862.

[59] Onkendi EM, Moleleki LN. Detection of *Meloidogyne enterolobii* in potatoes in South Africa and phylogenetic analysis based on intergenic region and the mitochondrial DNA sequences. Eu J Plant Pathol. 2013; 136: 1–5.

[60] Hazkani-Covo E, Zeller RM, Martin W. Molecular poltergeists: mitochondrial DNA copies (numts) in sequenced nuclear genomes. PLoS Genet. 2010; 6: 1000834 10.1371/journal.pgen.1000834 20168995 PMC2820518

[61] Parr RL, Maki J, Reguly B, Dakubo GD, Aguirre A, Wittock R, et al The pseudo-mitochondrial genome influences mistakes in heteroplasmy interpretation. BMC Genomics. 2006; 7: 185 16859552 10.1186/1471-2164-7-185 PMC1538596

[62] Hugall A, Moritz C, Stanton J, Wolstenholme DR. Low but strongly structured mitochondrial DNA diversity in root knot nematodes (*Meloidogyne*). Genetics. 1994; 136: 903–912. 7911772 10.1093/genetics/136.3.903 PMC1205895

[63] Castagnone-Sereno P, Danchin EGJ. Parasitic success without sex—the nematode experience. J Evol Biol. 2014; in press. 10.1111/jeb.12337 25105196

[64] Skantar AM, Carta LK, Handoo ZA. Molecular and morphological characterization of an unusual *Meloidogyne arenaria* population from Traveler´s tree *Ravenala madagascariensis* . J Nematol. 2008; 40: 179–189. 19440257 PMC2664674

[65] McClure MA, Nischwits C, Skantar AM, Schmitt ME, Subbotin SA. Root-Knot Nematodes in Golf course greens of the western United States. Plant Dis. 2012; 96: 635–347. 30727525 10.1094/PDIS-09-11-0808

[66] Maleita CM, Simoes MJ, Egas C, Curtis RHC, de O. Abrantes I. Biometrical, biochemical and molecular diagnosis of portuguese *Meloidogyne hispanica* isolates. Plant Dis. 2012; 96: 865–874. 30727353 10.1094/PDIS-09-11-0769-RE

[67] Tandingan De Ley I, De Ley P, Vierstraete A, Karssen G, Moens M, Vanfleteren J. Phylogenetic analyses of *Meloidogyne* small subunit rDNA. J Nematol. 2002; 34: 319–327. 19265950 PMC2620593

[68] Blok V, Powers T. Biochemical and molecular identification. In: Perry RN, Moens M, Starr J, editors. Root-knot nematodes. Lincoln; 2009. pp. 98–112.

[69] Lunt DH. Genetic test of ancient asexuality in root knot nematodes reveal recent hybrid origins. BMC Evol Biol. 2008; 8. 10.1186/1471-2148-8-194 PMC2478646 18606000

