# Supplementary material for: Comparative and Evolutionary Analyses of Meloidogyne spp. Based on Mitochondrial Genome Sequences
Source: PLoS One. 2015 Mar 23;10(3):e0121142. doi: 10.1371/journal.pone.0121142 (PMC4370701; doi:10.1371/journal.pone.0121142)

S1 Figure. Maximum Likelihood phylogenetic tree based on nucleotide sequences of eight mitochondrial protein-coding genes (4,920 bp). Numbers above branches represent bootstrap support values >50%. The mtDNA gene arrangement (GA) according to Liu *et al.* [8] is shown next to each species name.

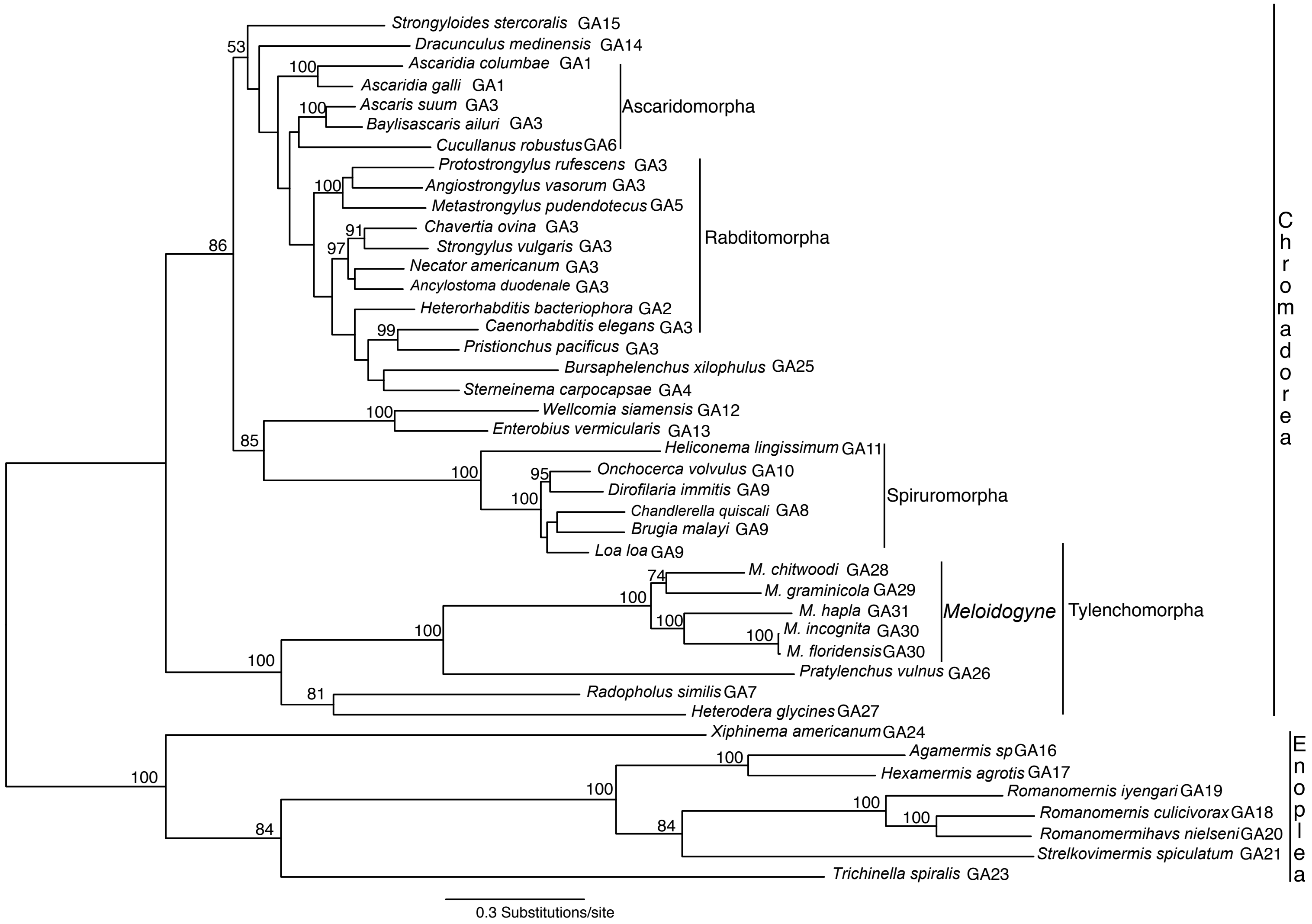

Supplement: S1 Fig — Numbers above branches represent bootstrap support values >50%. The mtDNA gene arrangement (GA) according to Liu et al. [8] is shown next to each species name. (PDF) [file pone.0121142.s001.pdf]
