# Supplementary material for: Comparative and Evolutionary Analyses of Meloidogyne spp. Based on Mitochondrial Genome Sequences
Source: PLoS One. 2015 Mar 23;10(3):e0121142. doi: 10.1371/journal.pone.0121142 (PMC4370701; doi:10.1371/journal.pone.0121142)

S2 Figure. Pairwise comparisons between the mtDNA of two strains of *M. incognita* (A) and two strains of *M. graminicola* (B), respectively, using the program VISTA. The Y-axis represents the sequence identity (50-100%). Protein (in violet) and rRNA (in yellow) genes are indicated in the standard nomenclature. tRNA genes (in red) are designated by a single-letter abbreviation.

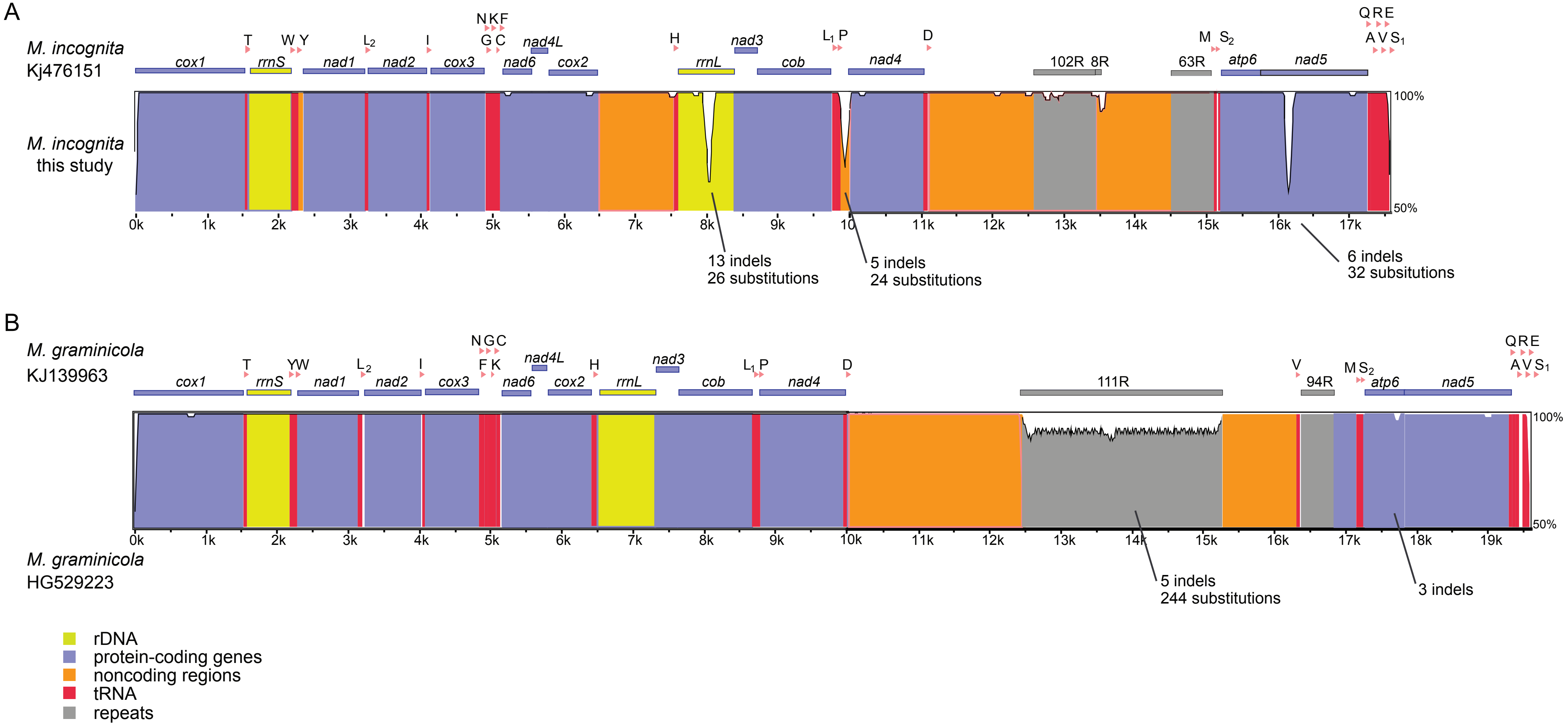

Supplement: S2 Fig — The Y-axis represents the sequence identity (50–100%). Protein (in violet) and rRNA (in yellow) genes are indicated in the standard nomenclature. tRNA genes (in red) are designated by a single-letter abbreviation. (PDF) [file pone.0121142.s002.pdf]
