# Supplementary material for: Comparative and Evolutionary Analyses of Meloidogyne spp. Based on Mitochondrial Genome Sequences
Source: PLoS One. 2015 Mar 23;10(3):e0121142. doi: 10.1371/journal.pone.0121142 (PMC4370701; doi:10.1371/journal.pone.0121142)

S3 Figure. Maximum Likelihood (ML) phylogenetic tree of *Meloidogyne* spp. based on the *cox2-rrnS* mitochondrial region. Numbers above branches represent ML bootstrap values >50%. Species names in red, green or black indicate species with mitotic parthenogenesis, meiotic parthenogenesis or unknown reproductive strategy, respectively.

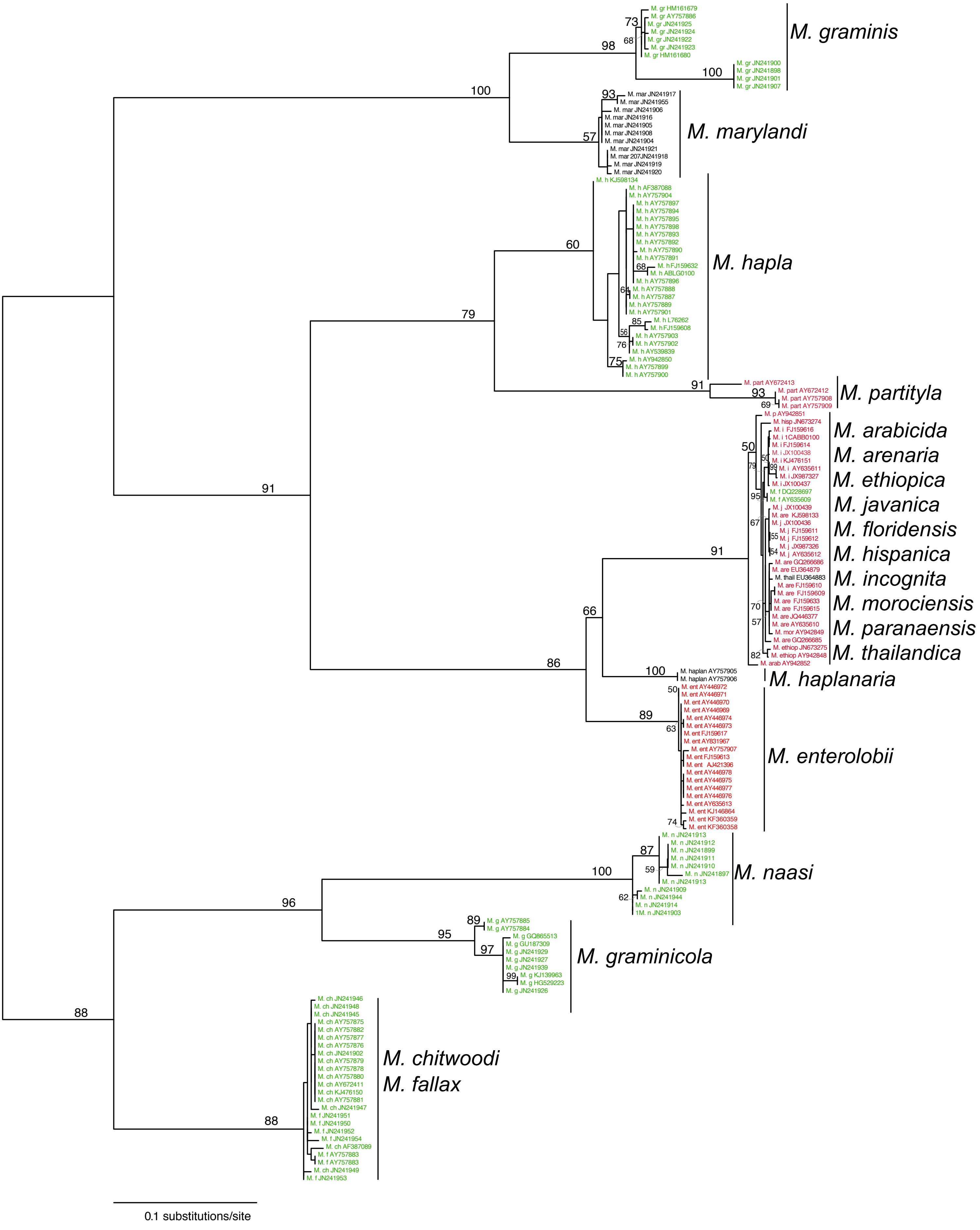

Supplement: S3 Fig — Numbers above branches represent ML bootstrap values >50%. Species names in red, green or black indicate species with mitotic parthenogenesis, meiotic parthenogenesis or unknown reproductive strategy, respectively. (PDF) [file pone.0121142.s003.pdf]
