# Supplementary material for: Comparative and Evolutionary Analyses of Meloidogyne spp. Based on Mitochondrial Genome Sequences
Source: PLoS One. 2015 Mar 23;10(3):e0121142. doi: 10.1371/journal.pone.0121142 (PMC4370701; doi:10.1371/journal.pone.0121142)
Supplement: S1 Table — (PDF) [file pone.0121142.s004.pdf]

S1 Table. GenBank accession numbers of *Meloidogyne* spp. with similarity to mitochondrial DNA.

M. floridensis	***M. hapla***	***M. incognita***
CCDZ01004464 (WGS)	ABLG01002664 (WGS)	CABB01003114 (WGS)
CCDZ01045455 (WGS)	BM902142 (EST)	CABB01002405 (WGS)
CCDZ01055118 (WGS)	ABLG01002088 (WGS)	CABB01006285 (WGS)
CCDZ01019919 (WGS)	BM884076 (EST)	
CCDZ01014978 (WGS)	ABLG01003195(WGS)	
CCDZ01030454 (WGS)		
CCDZ01023550 (WGS)		
CCDZ01013075 (WGS)		
CCDZ01049469 (WGS)		
CCDZ01012267 (WGS)		
CCDZ01021169 (WGS)		
AY635609 (nr/nt)		
