# Supplementary material for: Comparative and Evolutionary Analyses of Meloidogyne spp. Based on Mitochondrial Genome Sequences
Source: PLoS One. 2015 Mar 23;10(3):e0121142. doi: 10.1371/journal.pone.0121142 (PMC4370701; doi:10.1371/journal.pone.0121142)
Supplement: S2 Table — (PDF) [file pone.0121142.s005.pdf]

S2 Table. Taxonomic information and source of the *Meloidogyne* isolates included in this study.

Species name	Collection site	USDA Identification Code	GenBank accession number
M. arenaria	unknown	79H2	KM491199
M. arenaria	unknown	79H3	KM491200
M. arenaria	unknown	10C1	KM491201
M. arenaria	Palm Mulrooney	34B10	KM491202
M. arenaria	unknown	79H1	KM491203
M. arenaria	Mendoza, Argentina	NA	KM491204
M. chitwoodi	Tulelake, California (Isolate CAMC2)	NA	KJ476150
M. enterolobii	unknown	80D5	KM491197
M. enterolobii	unknown	80D4	KM491198
M. floridensis	Isolate 5	NA	CCDZ00000000
M. graminicola	Batangas, Philippines	NA	HG529223
M. graminicola	Hainan, China	NA	KJ139963
M. hapla	Netherlands	7J1	KM491205
M. hapla	Switzerland	6C1	KM491209
M. hapla	Netherlands	7J2	KM491210
M. hapla	California (strain VW9)	NA	ABLG01000000
M. incognita	Dominican Republic	66E4	KM491191
M. incognita	Dominican Republic	66E2	KM491192
M. incognita	Dominican Republic	66E3	KM491193
M. incognita	Nigeria	59B4	KM491189
M. incognita	Nigeria	59B3	KM491194
M. incognita	unknown	79F1	KM491195
M. incognita	unknown	79F2	KM491188
M. incognita	unknown	79F3	KM491190
M. incognita	Dominican Republic	66E1	KM491196
M. incognita	unknown	79G2	KM491207
M. incognita	unknown	79g1	KM491206
M. incognita	France (isolate NCMI4)	NA	KJ476151
M. incognita	Mexico (isolate Morelos)	NA	CABB01000000
M. naasi	Linn. Co. Oregon	12Al	KM491211
M. naasi	Linn. Co. Oregon	12A2	KM491211
M. naasi	Reading, FC	55F8	KM491213
M. naasi	Linn. Co. Oregon	12A4	KM491216
M. naasi	Linn. Co. Oregon	12A3	KM491214
M. naasi	Reading, FC	55K9	KM491215
M. naasi	unknown	79G3	KM491208
