# Supplementary material for: Comparative and Evolutionary Analyses of Meloidogyne spp. Based on Mitochondrial Genome Sequences
Source: PLoS One. 2015 Mar 23;10(3):e0121142. doi: 10.1371/journal.pone.0121142 (PMC4370701; doi:10.1371/journal.pone.0121142)
Supplement: S3 Table — (PDF) [file pone.0121142.s006.pdf]

S3 Table. Mitochondrial genome organization of *M. hapla, M. floridensis* and *M. incognita.*

Gene name	Range of identity (%) within the genus[a]	*M. hapla*			*M. floridensis*			*M. incognita*			Start/Stop codons						
		Gene length (in bp)	intergenic sequence[b]	%AT	Gene length (in bp)	intergenic sequence[b]	%AT	Gene length (in bp)	intergenic sequence[b]	%AT	*M. hapla*	*M. incognita*	*M. floridensis*	*M. incognita* [15]	*M. chitwoodi* [15]	*M. graminicola* [14]	*M. graminicola* [15]
cox1	81-99	1551	21	73.24	1557	-35	76.62	1557	-35	76.62	ATA/TAA	TTA/TAA	TTA/TAA	TTA/T	ATT/TAG	ATT	ATA/TAA
trnT	82-100	54	-1		55	-4		55	-4								
12S rRNA	78-99	616	-1	80.36	614	0	81.92	614	0	81.76							
trnY	81-100	54	2		53	15		53	15								
tRNA-W	77-100	53	-3		54	48		54	48								
nad1	72-99	861	-2	82.11	867	-15	83.51	867	-15	83.51	TTG/TAG	ATA/TAA	ATA/TAA	ATA/T	ATA/T	TTG/T	TTG/TAA
trnL	77-100	54	12		55	0		55	0								
nad2	67-99	nd	63	84.19	819	-15	89.87	819	-15	89.74	ATT/ND	ATT/TAA	ATT/TAA	ATT/T	ATT/TAA	ATT/TAA	ATA/TAA
trnI	78-100	55	35		54	4		54	4								
cox3	75-100	733	-5	80.51	762	-1	83.46	762	-1	83.46	GTT/TAA	ATA/TAA	ATA/TAA	ATA/TAA	ATT/T	TTG/TAA	TTG/TAA
trnN	78-100	55	-1		56	0		56	0								
trnG	85-100	53	-2		54	-2		54	-2								
trnK	83-100	55	-3		54	-4		54	-4								
trnC	85-100	51	-2		53	-4		53	-4								
trnF	87-100	54	3		54	-3		54	-3								
nad6	68-100	390	6	88.72	399	0	92.98	398	0	92.35	TTG/TAA	TTA/TAA	TTA/TAA	TTA/TAA	TTA/TAG	ATT/TAG	ATT/TAG
nad4L	68-99	228	-29	88.60	237	1	88.19	237	1	88.61	ATA/TAA	ATT/TAA	ATT/TAA	ATT/TAA	TTA/TAA	ATA/TAA	ATT/TAA
cox2	76-99	675	-2	78.97	693	535	80.84	693	1063	81.10	ATT/TAA	TTA/TAG	TTA/TAG	TTA/TAG	ATT/T	ATT/TAA	ATT/TAA
trn-H	79-100	55	0		54	-2		54	0								
16S rRNA	76-99	807	6	86.62	805	0	86.89	805	0	87.24							
nad3	70-100	306	16	89.22	315	17	88.89	315	17	88.89	ATT/TAG	TTA/TAG	TTA/TAG	TTA/TAG	ATT/TAG	TTG/TAG	ATT/TAG
cob	76-99	1029	4	80.56	1041	-15	82.61	1041	-15	82.52	ATG/TAA	ATA/TAA	ATA/TAA	ATA/T	TTA/TAA	ATA/TAG	ATA/TAG
trnL	77-100	56	-3		56	-3		56	-3								
trnP	84-100	54	18		54	-3		54	122								
nad4	76-99	1005	144	83.18	1173	3	84.74	1047	3	83.86	ATA/TAG	ATA/TAA	ATT/TAA	ATT/TAA	ATT/TAA	ATA/TAG	ATA/TAG
trnD	80-100	55	> 4596		54	>2774		54	4420-5910								
trnM	80-100	54	-1		54	3		54	3								
trnS	61-100	49	290		49	7		49	7								
atp6	66-99	561	6	84.31	552	3	88.04	552	3	88.04	ATG/TAG	ATA/TAA	ATA/TAA	ATA/TAA	ATT/TAA	ATT/TAA	TTT/TAA
nad5	67-99	1500	-15	83.67	1503	-15	84.96	1500	-15	84	ATA/TAA	TTA/TAA	TTA/TAA	TTA/T	TTA/TAA	ATA/TAA	ATA/TAA
trnQ	87-98	54	-3		54	0		54	0								
trnA	85-100	59	-8		53	0		53	0								
trnR	84-100	55	-5		55	-12		55	-12								
trnV	47-100	54	-3		55	-4		55	-4								
trnE	73-98	54	0		54	-1		54	-1								
trnS	77-98	49	74		49	19		49	19								

[a]Rrange of identity (%) among *M. floridensis, M. hapla, M. incognita, M. chitwoodi* and *M. graminicola*.

[b]Positive and negative values indicate gap nucleotides and overlapping nucleotides between two adjacent genes, respectively.
